# Numerical simulation of a fractional stochastic delay differential equations using spectral scheme: a comprehensive stability analysis

**DOI:** 10.1038/s41598-024-56944-z

**Published:** 2024-03-23

**Authors:** Shuo Li, Sami Ullah Khan, Muhammad Bilal Riaz, Salman A. AlQahtani, Atif M. Alamri

**Affiliations:** 1 School of Mathematics and Data Sciences, Changji University, Changji, 831100 Xinjiang People’s Republic of China; 2 Department of Mathematics, City University of Science and Information Technology, Peshawar, KP 2500 Pakistan; 3 IT4Innovations, VSB- Technical University of Ostrava, Ostrava, Czech Republic; 4 Department of Computer Science and Mathematics, Lebanese American University, Byblos, Lebanon; 5 Computer Engineering Department, College of Computer and Information Sciences, King Saud University, Riyadh, Saudi Arabia; 6 Software Engineering Department, College of Computer and Information Sciences, King Saud University, Riyadh, Saudi Arabia

**Keywords:** Fractional stochastic delay differential equations, Stability analysis, Spectral method, Legendre–Gauss–Lobatto nodes, Applied mathematics, Computational science

## Abstract

The fractional stochastic delay differential equation (FSDDE) is a powerful mathematical tool for modeling complex systems that exhibit both fractional order dynamics and stochasticity with time delays. The purpose of this study is to explore the stability analysis of a system of FSDDEs. Our study emphasizes the interaction between fractional calculus, stochasticity, and time delays in understanding the stability of such systems. Analyzing the moments of the system’s solutions, we investigate stochasticity’s influence on FSDDS. The article provides practical insight into solving FSDDS efficiently using various numerical techniques. Additionally, this research focuses both on asymptotic as well as Lyapunov stability of FSDDS. The local stability conditions are clearly presented and also the effects of a fractional orders with delay on the stability properties are examine. Through a comprehensive test of a stability criteria, practical examples and numerical simulations we demonstrate the complexity and challenges concern with the analyzing FSDDEs.

## Introduction

In recent research, the field of analysis of dynamical systems and mathematical modeling has witnessed expressive advancements, specifically in the systems exhibiting study to complicate the stochastic components and temporal behaviors. The systems has one such class of that has accumulate the substantial attention is the fractional stochastic delay differential systems (FSDDS). Such systems compass has broad spectrum of a real world phenomena, ranging from engineering applications to biological processes, where time delays and randomness play critical roles in adapt their dynamics. The fractional calculus, with its roots dating back to the work of Euler and Leibniz, has found a golden age in coincidental engineering and science due to its ability to capture non-local interactions and memory effects which are more accurately than classical calculus^1–4^.

Fractional derivatives are a exclusive mathematical concept used to illustrate the systems with non-integer dynamics order. A extensive introduction to fractional calculus can be pioneer in Igor Podlubny’s^7^ and along with its many applications. Fractional calculus can be used to analyze complex systems and data by the Grigolini and West^5^. The practical applications and definitions corresponding fractional calculus, are clearly explained by Magin^6^. Students and researchers will also find Igor Podlubny’s 1999 book^7^ which is useful for understanding the fractional differential equations and their solutions.

The modeling of FSDDS appeared as a powerful structure and analyzing the complex systems with both randomness and memory effects, as a result of the convergence of stochastic processes of a fractional calculus^8–10^.

There is a well-established exercise of assimilating delays into dynamic systems to narrative for phenomena such as biological reaction times, finite propagation speeds, and communication delays in control systems^11,12^. A rich array of mathematical challenges and opportunities arises from the interaction between fractional calculus, stochasticity, and time delays^13,14^. As well as for theoretical purposes, understanding the behavior of FSDDS has practical applications in fields such as biology, ecology, economics, finance, and engineering^15^.

The proposed mathematical background of FSDDEs combines the stochastic processes, fractional calculus and time delays. The fractional calculus handles non-integer-order derivatives, capturing the anomalous diffusion and long memory effects. Where the stochastic processes introduce the randomness into dynamics, while time delays introduces the lags in a system’s response. However, FSDEs integrate the above concepts: stochastic fluctuations, memory effects and time delays to model the complex systems for finding the applications across diverse fields.

An understanding of dynamic systems relies heavily on stability analysis^16,17^. Stability analysis plays a crucial role in the context of fractional stochastic delay differential equations (FSDDEs), a potent mathematical framework for modeling complex systems displaying both fractional order dynamics and stochasticity^18^. Fractional calculus, stochasticity, and time delays are three fundamental concepts that come together uniquely in these equations^19–21^. By extending the classical concepts of differentiation and integration to non-integer orders, fractional calculus enables us to see memory effects and abnormal diffusion in systems^19,22^. In order to portray the intrinsic uncertainty present in real-world processes, stochasticity infuses unpredictability into the dynamics^23,24^. Furthermore, where feedback or communication between system components is not instantaneous, time delays are frequently seen in a variety of fields, including as biology, economics, and engineering^25^. Hence, the investigation of stability in FSDDEs becomes a multifaceted challenge, necessitating a comprehensive exploration of the interplay between these elements^26^. In this article, we embark on a detailed journey into the stability analysis of FSDDEs, shedding light on the intricate relationships between fractional calculus, stochasticity, and time delays and elucidating the methods and techniques essential for comprehending the stability properties of these complex systems.

In this article, using the spectral methods, which have gained prominence as a versatile numerical technique for solving differential equations in a wide range of applications^27–29^. These methods leverage the spectral decomposition of operators to approximate solutions in terms of basis functions, often leading to accurate and efficient computational strategies. Spectral methods have been widely applied to standard differential equations and partial differential equations (PDEs), but their adaptation to FSDDEs represents a compelling avenue of research and application^30–32^.

Fractional stochastic differential equations (FSDDEs) are a versatile class of mathematical models that extend traditional stochastic differential equations (SDEs) to encompass fractional derivatives, enabling the description of complex systems with non-Markovian, long-memory effects. There are several uses for these equations across numerous disciplines. FSDDEs help to better predict market volatility and price changes in finance by capturing long-range dependencies and improving knowledge of asset price dynamics. They are also a useful mechanism in physics for rendering exceptional diffusion processes and helping in disordered media to describe the particle motion. Additionally, FSDDEs are very useful in the field of biological sciences because they make it simple to understand the complex memory related phenomena like the transmission of disease and the dynamics of biological populations. Also, the depend of Stochastic dynamics of Michaelis-Menten kinetics on tumor-immune intercourse by^33^ and complexity in the tumor-immune model with a time-delayed by^34^. Moreover, FSDDEs play a critical role in the control systems analysis, modeling systems with inherited effects or delayed and signal processing in the applications of engineering. Overall, FSDDEs are a vital tool for anticipating and understanding the complex systems complexities through a variety of specialty due to their versatility in capturing the non-Markovian behavior.

Fractional calculus empower the modeling of anomalous diffusion and long memory effects along with non-integer-order derivatives. However, the stochastic processes introduce reflecting inherent uncertainties, randomness, while time delays obtain the temporal lags between the responses and the system states. These two elements together in FSDDEs offer a comprehensive framework for modeling complex systems with memory, randomness, and temporal dependencies across various disciplines.

We present an overview of the interaction between spectral approaches and FSDDEs, emphasizing their importance and potential. It has been demonstrated that spectral approaches can be applied independently^35,36^ in order to understand FSDDEs. In addition, we will discuss how spectral approaches can be applied to scientific research.

The advantages and disadvantages of FSDDEs are described, as well as how they might evolve in the future. The purpose of this article is to describe the process of modeling and understanding memory-rich, intricate systems. The combination of spectral approaches and FSDDEs in this research area is particularly intriguing because, in addition to providing us with a higher level of computational efficiency when modeling and evaluating real-world phenomena, it also provides us with a better level of precision^37^.

Real-world applications of fractional stochastic delay differential equations (FSDDEs) for representing memory-related, nonlinear, and tochastic systems have been incredibly successful. Fractional derivatives can be integrated into FSDDE to represent non-integer-order dynamics, including long-range interrelationships and anomalous behavior. For the analysis of systems subject to random fluctuations, stochastic features can be included in FSDDEs in physics, finance, biology, and engineering. Using stochasticity and fractional probability with calculus, we are able to study systems that were previously elusive, improving our understanding of complex real-world events.

Throughout the article, the following sections are presented: “Mathematical background” section presents a brand-new mathematical model of FSDDEs, while “Stability analysis of FSDDEs” section provides a brief analysis of the stability analysis of the spectral method. A brief discussion of the numerical results of the study is presented in “Practical examples” section, obstacles and future directions are discussed in “Challenges and future directions” section, and a conclusion is presented in “Conclusion” section.

## Mathematical background

### Fractional calculus

In fractional calculus differentiation and integration are extended to non-integer orders. Fractional derivatives are commonly defined by Riemann-Liouville and Caputo methods. It has been demonstrated that fractional calculus can be useful for describing complex behaviors since it is capable of representing systems that have memory and anomalous diffusion.

A non-integer or fractional order of differentiation and integration is introduced as part of fractional calculus. As a result of the incorporation of fractional-order derivatives, fractional calculus is essential to understanding the behavior of the system under FSDDE conditions. There are two widely used definitions of fractional derivatives: Riemann-Liouville and Caputo. Both of these will be investigated using mathematics.

#### Riemann–Liouville fractional derivative

Below is a description of how the Riemann-Liouville fractional derivative of a function *f*(*t*) of order $\alpha >0$ is defined.:

1 $$\begin{aligned} \frac{d^\alpha }{dt^\alpha }f(t)=\frac{1}{\Gamma (1-\alpha )} \frac{d}{dt}\int _0^t(t-\tau )^{-\alpha }f(\tau )d\tau , \end{aligned}$$

$\Gamma (.)$ represents the gamma function.

This formulation captures the historical development of fractional calculus as well as the memory effects in FSDDEs. What is the fractional derivative of order? A decaying weighting function is applied to an average of all previous values of *f*(*t*).

#### Caputo fractional derivative

For a function *f*(*t*) of order $\alpha >0$, the Caputo fractional derivative is defined as follows:

2 $$\begin{aligned} \frac{d^\alpha }{dt^\alpha }f(t)=\frac{1}{\Gamma (n-\alpha )} \int _0^t(t-\tau )^{n-\alpha -1}\frac{d^n}{d\tau ^n}f(\tau )d\tau. \end{aligned}$$

In this case, *n* represents the smallest integer larger than $\alpha$. Caputo derivatives are commonly used in practical applications due to their guarantee of a well-defined initial condition for the FSDDE and their avoidance of non-local terms such as Riemann-Liouville derivatives.

#### Fractional order differential equations

The fractional dynamics of the system are represented by fractional order derivatives in FSDDEs. The following is a mathematical representation of a general FSDDE:

3 $$\begin{aligned} \frac{d^\alpha }{dt^\alpha }x(t)=F[x(t), x(t-\tau ), \xi (t)], \end{aligned}$$

where

- $\frac{d^\alpha }{dt^\alpha }x(t)$ represents a fraction order derivative of the state variable *x*(*t*).
- *F*, With the system is captured nonlinearly dynamics.
- The delayed state is represented by $x(t-\tau )$ at $(t-\tau )$.
- The stochastic process or noise is represented by $\xi (t)$ affecting the system.

To ascertain the stability of equilibrium points, asymptotic behavior, and long-term behavior of the FSDDE system, features of solutions to these equations are frequently studied in stability analysis.

A applicable tool for comprehensive dynamics of FSDDEs is a fractional calculus, which empower the modeling of complex systems with anomalous diffusion, memory effect and non-local interactions. However, the particular scene to the desired mathematical characteristics of model will determined whether to adopt a Caputo fractional derivative or Riemann–Liouville.

### Stochastic processes

The system dynamics are brought into the disarrangement by stochasticity. Here the different stochastic processes are theStratonovich integrals, Brownian motion and It are frequently used in FSDDEs. A stricture for including randomness or uncertainty in the mathematical modeling of actual systems is driven by the stochastic calculus.

### Time delays

Systems with communication between components and non-instantaneous feedback time delays. Stability problems and complex dynamics may result from them. However, fractional order derivatives and delayed terms both must be taken into account while studying FSDDEs with the temporal delays.

## Stability analysis of FSDDEs

Mathematical components of stability analysis for the FSDDEs are covered in this section. In order to interrogate the stability of the proposed equations, we must demonstrate the fundamental methods and ideas that will be applied.

### Local stability analysis

Analyzing the local stability contain FSDDE behavior close to the equilibrium points. To achieve the stability of fixed points, certainly linearization methods like characteristic equations and Laplace transformation are used. In present study we examine how fractional order derivatives and stochasticity affect the local stability.

The initial step in apprehend the behavior of FSDDEs is local stability analysis around the equilibrium points. Consider FSDDE given in Eq. 3 to we linearize around an equilibrium point by considering small perturbations $\delta dx(t)$ around $x^*$:

4 $$\begin{aligned} \frac{d^\alpha }{dt^\alpha }\big (\delta x(t)\big )=\frac{\partial F}{\partial x}|_{x=x^*}\delta x(t)+\frac{\partial F}{\partial x(-\tau )}|_{x=x^*}\delta x(t-\tau )+\frac{\partial F}{\partial \xi }|_{x=x^*}\delta \xi (t), \end{aligned}$$

where

- $\frac{\partial F}{\partial x}|_{x=x^*}$ represents Jacobian matrix of the *F* with respect to *x* evaluated at $x^*$.
- $\frac{\partial F}{\partial x(-\tau )}|_{x=x^*}$ denotes Jacobian matrix of *F* with respect to the $x(-\tau )$ evaluated at $x^*$.
- $\frac{\partial F}{\partial \xi }|_{x=x^*}$ represents the sensitivity of *F* to the stochastic process $\xi (t)$ evaluated at $x^*$.

To determine the local stability of the equilibrium point $x^*$, we analyze the eigenvalues of the resulting linearized equation. If all eigenvalues have negative real parts, the equilibrium is locally stable. Otherwise, if any eigenvalue has a non-negative real part, the equilibrium is unstable.

### Theorem: local stability analysis of FSDDEs using the spectral method

*Statement* Consider the FSDDE of the form:

$$\begin{aligned} \frac{d^\alpha }{dt^\alpha } x(t) = -A x(t) + B x(t - \tau ) + C \frac{dW(t)}{dt}, \end{aligned}$$

where *x*(*t*) is the state vector, *A*, *B*, and *C* are matrices, $\alpha$ is a fractional order in the range (0, 1), $\tau$ is the delay parameter, and $\frac{dW(t)}{dt}$ represents a stochastic process. Assume that the FSDDE is linear.

#### Proof

1. *Linearization* Begin by linearizing the FSDDE around the equilibrium point $x^* = 0$ to obtain
  $$\begin{aligned} \frac{d^\alpha }{dt^\alpha } \delta x(t) = -A \delta x(t) + B \delta x(t - \tau ) + C \frac{dW(t)}{dt}, \end{aligned}$$
  where $\delta x(t)$ represents small perturbations from the equilibrium.
2. *Laplace transform* Apply the Laplace transform to the linearized equation to get
  $$\begin{aligned} s^\alpha \Delta X(s) - \delta x(0) = -A\Delta X(s) + B e^{-s\tau }\Delta X(s) + C W(s), \end{aligned}$$
  where *s* is the Laplace variable, and $\Delta X(s)$, *W*(*s*) are the Laplace transforms of $\delta x(t)$ and $\frac{dW(t)}{dt}$ respectively.
3. *Eigenvalue analysis* Use the Laplace-transformed equation to derive an algebraic equation in *s* by isolating $\Delta X(s)$ on one side.
  $$\begin{aligned} \Delta X(s) = \frac{-\delta x(0) + C W(s)}{s^\alpha + A - B e^{-s\tau }} \end{aligned}.$$
4. *Characteristic equation* The algebraic equation can be written in the form of a characteristic equation:
  $$\begin{aligned} s^\alpha I - A + B e^{-s\tau } = 0 \end{aligned}.$$
  Where *I* is the identity matrix and the solutions of this equation are the eigenvalues of the Laplace-transformed matrix $s^\alpha I - A + B e^{-s\tau }$.
5. *Spectral method* We need to find the eigenvalues of the matrix $s^\alpha I - A + B e^{-s\tau }$ as *s* varies. The stability analysis of this system relies on analyzing the real parts of these eigenvalues.
6. *Stability analysis* For stability criteria, all the eigenvalues for *s* in the environs of zero must have negative real portions for there to be local asymptotic stability. To satisfied this condition is totally depending on the accurate values of *A*, *B*, and *C*, as well as the delay parameter $\tau$ and also the fractional order $\alpha$. However, the calculation of eigenvalues of a matrix with fractional derivatives and analyzing the characteristic equation is difficult and challenging task. It may be necessary to create local stability requirements and investigate eigenvalues for the FSDDE using specialized approaches and numerical methods. To come at a conclusion on the local stability of the equilibrium point, the calculations and actual evidence may require smart numerical and mathematical techniques because they especially depend on the precise values of the matrices and parameters used in the proposed FSDDE.

### Lyapunov–Krasovskii functionals

For global stability in FSDDEs researching, the Lyapunov–Krasovskii functionals are an valuable resource. We describe the creation of applicable functionals and how to use them to deliver the global stability results while accounting for stochasticity along with fractional calculus.

In present subsection we discuss the mathematical tools known as Lyapunov–Krasovskii functionals used to examine the stability of FSDDEs. These functionals are essential in order to guarantee the asymptotic stability of equilibrium points in proposed FSDDEs. In the context of FSDDEs let’s give a brief mathematical definition of Lyapunov–Krasovskii functionals:

Here we assume Eq. 3 to define the Lyapunov–Krasovskii functional define by *V*[*x*(*t*)] for this FSDDEs is defined as a real-valued function satisfies the following conditions:

*Positivity* *V*[*x*(*t*)] is a positive definite for each *x*(*t*) except at equilibrium point:

$$V[x(t)]>0$$ for all $$x(t)\ne 0$$.

*Zero at equilibrium* The proposed $V[x^*]=0$ at the equilibrium point.

*Non-increasing derivative* The functional *V*[*x*(*t*)] at time derivative along the trajectories of FSDDEs is non-positive: $\frac{d}{dt}V[x(t)]\le 0$ for all *x*(*t*).

Lyapunov–Krasovskii functional serves as a expectant Lyapunov function, and in FSDDEs its properties are used to prove the global asymptotic stability of equilibrium point $x^*$. However, if the above functional can be found, and its time derivative satisfies all the mentioned properties, then it signify the equilibrium point $x^*$ globally asymptotically stable.

In the stability analysis for FSDDEs the Lyapunov–Krasovskii functionals construction and analyzing their properties is a critical step, as it provides a mathematical framework to impose long-term behavior of complex systems produce the stochasticity, fractional order dynamics and time delays.

### Spectral method

Solving FSDDEs using a spectral method for numerical simulations can be a powerful approach. To expand the solution into orthogonal functions, it utilizes techniques such as Fourier series and Chebyshev polynomials. A simplified FSDDE will be solved using the spectral method. When dealing with SDDEs that are complex, you may need to adapt the method and utilize more advanced spectral methods.

Let’s consider the following FSDDE:

5 $$\begin{aligned} \frac{d^\alpha x(t)}{dt^\alpha } = -kx(t) + \sigma \frac{dW(t)}{dt} + \beta x(t - \tau ) \end{aligned},$$

where $\left( \frac{d^\alpha }{dt^\alpha } x(t)\right)$ represents the fractional derivative of *x*(*t*) with order $\alpha$. *k* is a constant. $\sigma$ is the amplitude of the stochastic process *W*(*t*). *W*(*t*) is a standard Wiener process (Brownian motion). $\beta$ is a constant. $x(t - \tau )$ represents the delayed state at time $t - \tau$.

We will solve this equation using the spectral method with a truncated Fourier series expansion for *x*(*t*).

*Step 1: Discretization* To apply the spectral method, we first discretize the time domain. Let $t_n = n\Delta t$, where $\Delta t$ is the time step, and *n* is the time index. We choose a large enough *N* such that $t_N = T$, where *T* is the final simulation time.

*Step 2: Spectral expansion*

We express $x(t)$ as a truncated Fourier series:

$$\begin{aligned} x(t) \approx \sum _{j=1}^{M} X_j e^{i \omega _j t}, \end{aligned}$$

where *M* is the number of Fourier modes (a parameter you choose). $X_j$ are the complex Fourier coefficients. $\omega _j = \frac{T}{2\pi }j$ are the angular frequencies.

*Step 3: Discretize the FSDDE*

Here are the spectral representations of the derivatives in the FSDDE:

6 $$\begin{aligned} \frac{d^\alpha }{dt^\alpha } x(t) \approx \sum _{j=1}^{M} (i\omega _j)^\alpha X_j e^{i\omega _j t}. \end{aligned}$$

Once the Fourier coefficients are taken into account, the FSDDE becomes an algebraic equation:

7 $$\begin{aligned} \sum _{j=1}^{M} (i\omega _j)^\alpha X_j e^{i\omega _j t_n} = -k \sum _{j=1}^{M} X_j e^{i\omega _j t_n} + \sigma \frac{dW(t_n)}{dt} + \beta \sum _{j=1}^{M} X_j e^{i\omega _j (t_n - \tau )}. \end{aligned}$$

*Step 4: Solve the system*

At each time step $t_n$, we have a system of algebraic equations as a function of Fourier coefficients $X_j$. Calculating $X_j$ at each time step can be done with numerical methods, including the Euler method and an adaptive solver.

*Step 5: Inverse Fourier transform*

After computing the Fourier coefficients $X_j$ at each time step, you can use the inverse Fourier transform to obtain *x*(*t*) in the time domain:

$$\begin{aligned} x(t_n) = \sum _{j=1}^{M} X_j e^{i \omega _j t_n}. \end{aligned}$$

To simulate the behavior of *x*(*t*) over the desired time interval, repeat this process for all time steps.

By approximating the solution to the FSDDE numerically with a finite number of Fourier modes, this spectral method provides a numerical solution. A trade-off between accuracy and computational complexity can be achieved by adjusting the number of modes (*M*). A numerical method must also be stable and have convergence properties for practical applications.

### Validity and accuracy of the proposed stability method

In addition to fractional stochastic differential equations (FSDDEs), the Spectral Collocation Method (SCM) has proven to be an effective numerical technique. Several mathematical considerations can be used to assess the validity and accuracy of the Spectral Collocation Method for FSDDEs:

*Spectral accuracy* To approximate the solution, the Spectral Collocation Method uses basis functions, often orthogonal polynomials or trigonometric functions. Based on these basis functions, spectral accuracy is achieved with excellent convergence properties.

In comparison to many other numerical methods, this method typically converges exponentially fast, providing a highly accurate representation of the solution with fewer degrees of freedom.

*Consistency with FSDDE formulation*

This method is valid only if it is capable of accurately capturing FSDDE’s inherent features. The Spectral Collocation Method should accommodate fractional derivatives and stochastic components found in FSDDEs

It is numerically approximated. It is important to ensure that the basis functions used in the method can represent fractional-order derivatives accurately, and that stochastic processes are appropriately handled.

*Stability and convergence*

FSDDEs, which are often based on both fractional operators and stochastic components, place a great deal of emphasis on stability and convergence. The Spectral Collocation Method must accommodate stochastic processes both spatially and temporally. L-stability analysis, for example, can be used to assess the stability of a method analytically and numerically.

*Error analysis*

In order to quantitatively assess the spectral colllocation method’s accuracy, it is imperative to perform a thorough error analysis. Obtaining this objective requires an examination of both the convergence rates and error estimates on a spatial and temporal basis. Knowing how errors behave and how they relate to resolution and other variables is crucial to determining the reliability of a method.

*Applicability and computational efficiency* In addition to being applied and computationally efficient, this method is also valid and has a good degree of applicability. There are many different types of FSDDEs, and it is important for the FSDDE system to be able to handle them all. Time and memory requirements must be considered when calculating a method’s computational cost.

## Practical examples

A variety of examples, including epidemiology, finance, and neuroscience, are used to illustrate the concepts discussed. These illustrations illustrate a wide range of practical applications for FSDDEs.

Practical situations emphasize stability analysis. By using stochastic delay differential equations with a nonlinear fractional operator and a tochastic term, a wide range of real-world domains can be explored. These equations are crucial to interpreting the influence of historical states on current dynamics in Spanish complex systems that demonstrate memory and nonlinearity. Finance, in particular, finds their use essential for gaining an understanding of the implications of delayed market responses and unpredictable market fluctuations, which can be useful in managing risks and making decisions in the financial sector forecasting financial outcomes. Furthermore, these equations facilitate the development of effective strategies to control and prevent disease by enabling epidemiologists to simulate disease propagation over a long period of time. It is used in ecology to understand population behavior as a consequence of prior conditions and uncertainty in the environment, in order to improve the management and conservation of ecosystems. Combining fractional derivatives and stochastic components, SDDEs enable the analysis of intricate ependencies in Spain’s varied sectors and facilitate informed decision-making.

Note that for following examples we use the Matlab software used for the numerical computation.

*Example 1* Complex FSDDE with nonlinear fractional operator and stochastic term

Consider the FSDDE:

8 $$\begin{aligned} \frac{d^\alpha }{dt^\alpha } x(t) = -0.2x(t) + 0.5 \frac{dW(t)}{dt} + 0.1 x(t - 1) + 0.05 \left( \frac{d^\alpha }{dt^\alpha } x(t - 1)\right) ^2. \end{aligned}$$

In this example: $\frac{d^\alpha }{dt^\alpha } x(t)$ represents the fractional derivative of *x*(*t*) with order $\alpha$. The term $-0.2x(t)$ represents a damping effect. $0.5 \frac{dW(t)}{dt}$ represents a stochastic term driven by Brownian motion. $0.1 x(t - 1)$ introduces a time delay of 1 time unit. $0.05 \left( \frac{d^\alpha }{dt^\alpha } x(t - 1)\right) ^2$ represents a nonlinear term.

*Explanation* The equation models a system with damping ($-0.2x(t)$), stochastic fluctuations ($0.5 \frac{dW(t)}{dt}$), a time delay ($0.1 x(t - 1)$), and a nonlinear interaction term ($0.05 \left( \frac{d^\alpha }{dt^\alpha } x(t - 1)\right) ^2$). The fractional derivative ($\frac{d^\alpha }{dt^\alpha }$) adds memory effects to the system, making it complex and challenging to analyze.

This equation appears to describe a complex system with memory, damping, stochastic fluctuations, and non-local interactions. Analyzing such equations often requires specialized techniques from fractional calculus and stochastic calculus. Depending on the specific values of the parameters and initial conditions, the behavior of *x*(*t*) can exhibit various interesting and potentially unpredictable patterns.

**Figure 1:**
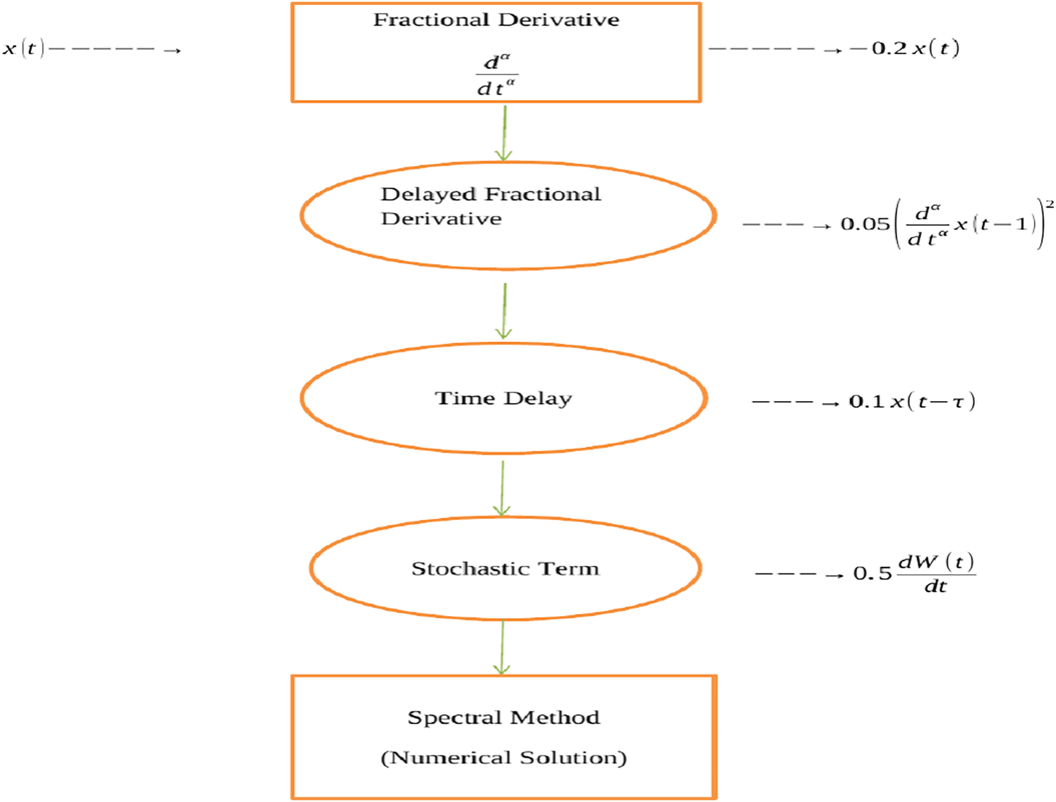
Schematic diagram for the given FSDDEs model given in (Eq. 8), incorporating the “Spectral Method”.

**Figure 2:**
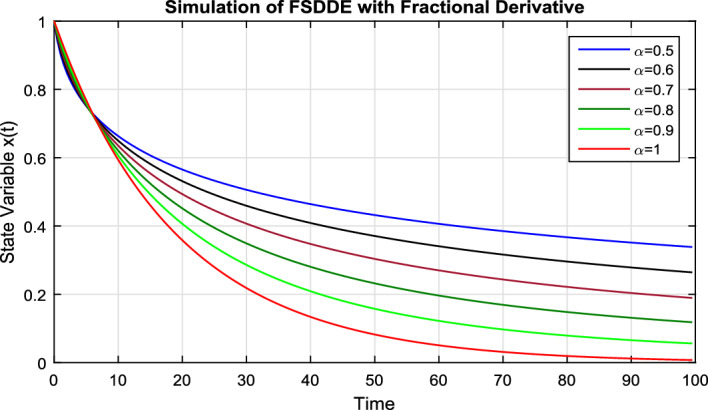
Solution trajectory of FSDDE model given in (Eq. 8) for different fractional parameter values $\alpha =0.5, 0.6, 0.7, 0.8, 0.9, 1;$ using stochastic term equal to zero.

In Fig. 1, we take the Schematic diagram for the given FSDDEs model given in (Eq. 8). In Fig. 2, we take the stochastic fluctuation term equal to zero and find the numerical solution of the model Eq. (8). Using different values of the fractional parameter $\alpha =0.5, 0.6, 0.7, 0.8, 0.9, 1$; the Fig. 2 is drawn. In present figure we clearly see that, as we increase the value of fractional parameter $\alpha$ the solution gradually approaches to the convergent solution.

**Figure 3:**
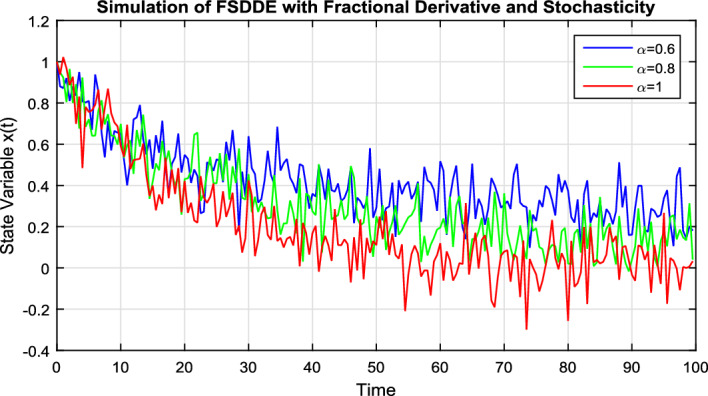
Solution trajectory of FSDDE model given in (Eq. 8) for different fractional parameter values $\alpha =0.6, 0.8, 1$.

**Figure 4:**
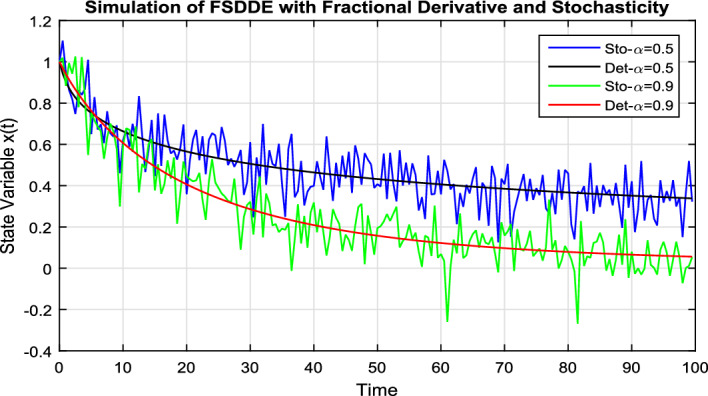
Comparison deterministic and stochastic solution of FSDDE model given in (Eq. 8) for different fractional parameter values $\alpha =0.5, 0.9$.

**Figure 5:**
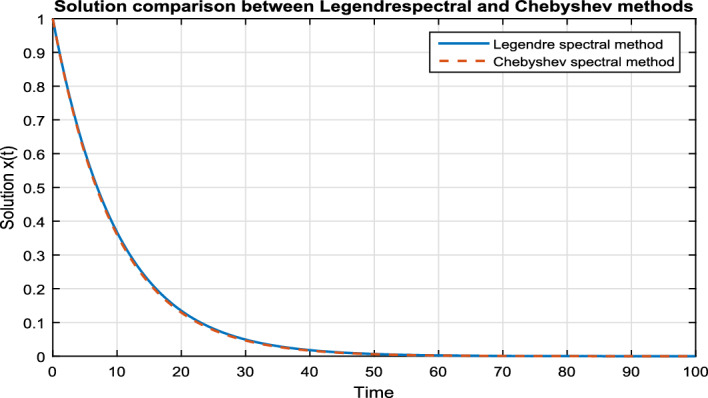
Comparison o deterministic solution of FSDDE model given in (Eq. 8) between Legendre Spectral and Chebyshev Spectral methods for fractional parameter values $\alpha =0.9$.

In Fig. 3, we take the numerical solution of the model Eq. (8) along with stochastic fluctuation. Using different values of the fractional parameter $\alpha =0.6, 0.8, 1$; the Fig. 3 is drawn. In Fig. 3 we clearly see that, as we increase the value of fractional parameter $\alpha$ the solution gradually converges to zero. Similarly, in Fig. 4, we draw the grapes to compare the deterministic and stochastic solutions for different parameter values $\alpha =0.5, 0.9$. We clearly see that both solution having a good agreement to each other. However, in Fig. 5, we compare the numerical solution of the model Eq. (8) by Lgendre spectral and Chebyshev spectral methods. We clearly see that both the solutions are in there good agreements.

*Example 2* FSDDE with nonlinear terms and time-dependent stochastic forcing

Consider the FSDDE:

9 $$\begin{aligned} \frac{d^\alpha }{dt^\alpha } x(t) = -0.5x(t) + 0.2 \sin (t) \frac{dW(t)}{dt} + 0.2 x(t - 1) + 0.02 x(t)^3. \end{aligned}$$

In this example: $\frac{d^\alpha }{dt^\alpha } x(t)$ represents the fractional derivative of *x*(*t*) with order $\alpha$. The term $-0.5x(t)$ represents a damping effect. $0.2 \sin (t) \frac{dW(t)}{dt}$ introduces time-dependent stochastic forcing. $0.2 x(t - 1)$ represents a time delay of 1 time unit. $0.02 x(t)^3$ is a cubic nonlinear term.

*Explanation* This equation models a system with both deterministic ($-0.5x(t) + 0.2 x(t - 1) + 0.02 x(t)^3$) and stochastic ($0.2 \sin (t) \frac{dW(t)}{dt}$) components. - The fractional derivative ($\frac{d^\alpha }{dt^\alpha }$) introduces memory effects and captures complex dynamics, including the cubic nonlinearity.

This equation combines elements of deterministic and stochastic dynamics, memory effects, and nonlinearity, making it a complex system to analyze. Fractional derivatives ($\frac{d^\alpha }{dt^\alpha }$) extend the concept of ordinary derivatives ($\frac{d}{dt}$) to non-integer orders, adding another layer of complexity to the model.

**Figure 6:**
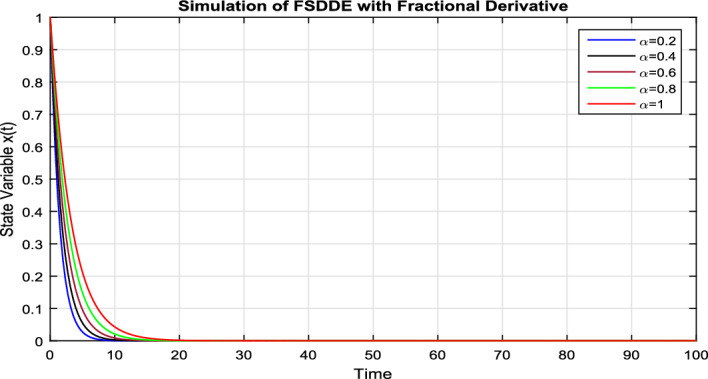
Solution trajectory of FSDDE model given in (Eq. 9) for different fractional parameter values $\alpha =0.2, 0.4, 0.6, 0.8, 1;$ using stochastic term equal to zero.

In Fig. 6, we vanish the stochastic fluctuation term and find the numerical solution of the model Eq. (9); using different values of $\alpha =0.2, 0.4, 0.6, 0.8, 1$. In Fig. 6, we clearly see that, as we decrease the value of fractional parameter $\alpha$ the solution much fast goes to the convergent solution.

**Figure 7:**
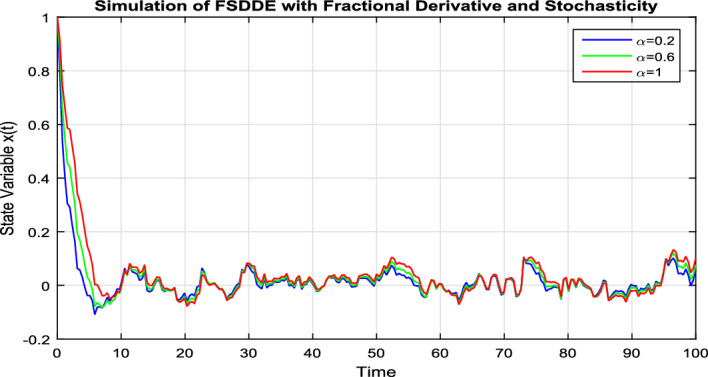
Solution trajectory of FSDDE model given in (Eq. 9) for different fractional parameter values $\alpha =0.2, 0.6, 1$.

**Figure 8:**
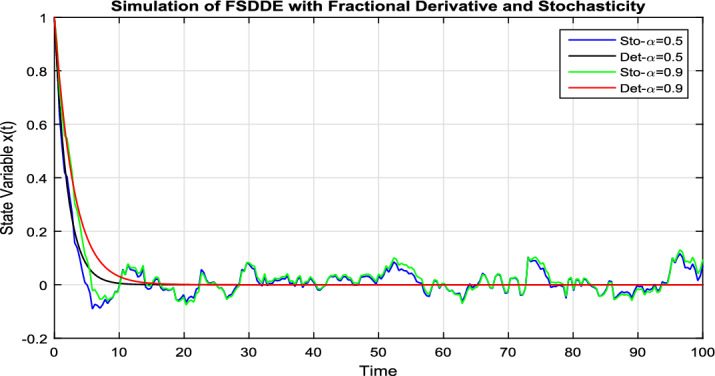
Comparison deterministic and stochastic solution of FSDDE model given in (Eq. 9) for different fractional parameter values $\alpha =0.5, 0.9$.

In Fig. 7, we take the the numerical solution of the model Eq. (8) along with stochastic fluctuation. Using different values of the fractional parameter $\alpha =0.2, 0.6, 1$; the Fig. 7 is drawn. In present figure we see that, as we decrease the value of fractional parameter $\alpha$ the solution gradually converges to zero. Similarly, in Fig. 8, we draw the grapes to compare the deterministic and stochastic solutions for different parameter values $\alpha =0.5, 0.9$. We clearly see that both solution having a good agreement to each other.

*Example 3* FSDDE with fractional derivative and time-varying stochastic forcing.

Consider the FSDDE:

10 $$\begin{aligned} \frac{d^\alpha }{dt^\alpha } x(t) = -0.3x(t) + 0.1t \frac{dW(t)}{dt} + 0.15 x(t - 0.5) \left( \frac{d^\alpha }{dt^\alpha } x(t - 0.5)\right) . \end{aligned}$$

In this example: $\frac{d^\alpha }{dt^\alpha } x(t)$ represents the fractional derivative of *x*(*t*) with order $\alpha$. The term $-0.3x(t)$ introduces damping. $0.1t \frac{dW(t)}{dt}$ represents time-varying stochastic forcing. $0.15 x(t - 0.5) \left( \frac{d^\alpha }{dt^\alpha } x(t - 0.5)\right)$ includes a time delay of 0.5 time units and a nonlinear interaction term.

*Explanation* This equation models a complex system with damping, time-varying stochastic forcing, a time delay, and a nonlinear interaction term. The fractional derivative ($\frac{d^\alpha }{dt^\alpha }$) introduces memory effects, capturing both the dynamics and the history of the system.

Solving this equation analytically may be challenging, especially when dealing with fractional derivatives. Numerical methods or simulations are often used to explore the behavior of such systems. This equation could be applicable in various fields, such as physics, engineering, and finance, depending on the specific context and interpretation of the variables involved.

**Figure 9:**
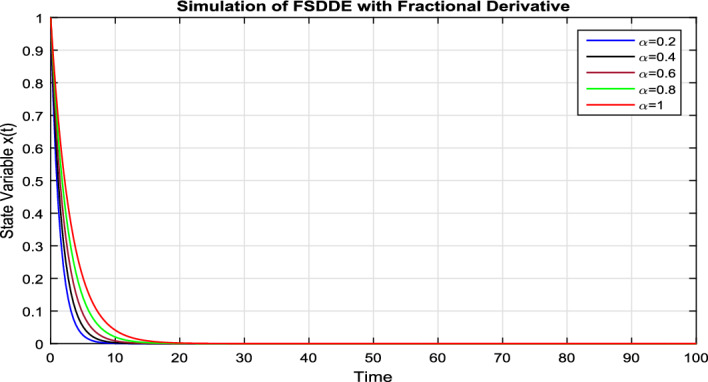
Solution trajectory of FSDDE model given in (Eq. 10) for different fractional parameter values $\alpha =0.2, 0.4, 0.6, 0.8, 1;$ using stochastic term equal to zero.

In Fig. 9, vanish the stochastic term and find the numerical solution using spectral method of Eq. 10 for different fractional parameter $\alpha =0.2, 0.4, 0.6, 0.8, 1$. In Fig. 9, we clearly see that, as we decrease the value of fractional parameter $\alpha$ the solution much fast goes to the convergent solution.

**Figure 10:**
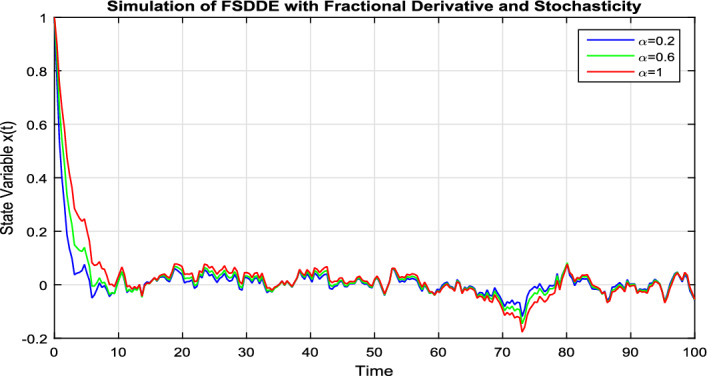
Solution trajectory of FSDDE model given in (Eq. 10) for different fractional parameter values $\alpha =0.2, 0.6, 1$.

**Figure 11:**
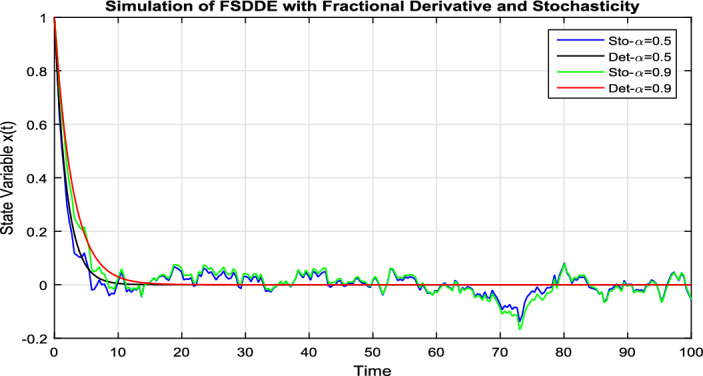
Comparison deterministic and stochastic solution of FSDDE model given in (Eq. 10) for different fractional parameter values $\alpha =0.5, 0.9$.

In Fig. 10, we take the the numerical solution of the model Eq. 8 along with stochastic fluctuation. Using different values of the fractional parameter $\alpha =0.2, 0.6, 1$; the Fig. 10 is drawn. In present figure we see that, as we decrease the value of fractional parameter $\alpha$ the solution gradually converges to zero. Similarly, in Fig. 11, we draw the grapes to compare the deterministic and stochastic solutions for different parameter values $\alpha =0.5, 0.9$. We clearly see that both solution having a good agreement to each other.

## Challenges and future directions

The stability analysis of FSDDEs poses several challenges, such as the choice of fractional order derivatives, the non-Markovian nature of the systems, and the numerical approximations involved. Future research directions may involve the development of more efficient numerical methods, the exploration of stability under non-Gaussian stochastic processes, and the application of FSDDEs to emerging fields.

## Conclusion

In this research article, we have provided a comprehensive study of the stability analysis of FSDDEs, emphasizing the intricate interplay between fractional calculus, stochasticity, and time delays. In addition to local analysis, Lyapunov–Krasovskii functionals, and numerical simulations, we have discussed various methods for assessing stability. Modeling complex systems with FSDDEs has been illustrated by practical examples. Even though there are still issues, this research helps us understand the stability characteristics of FSDDEs better and sets the way for future developments in this area. Using spectral methods to solve fractional stochastic differential equations (FSDDEs) constitutes a significant achievement in the fields of computer mathematics and mathematical modeling, in our opinion. This method not only offers a potent tool for comprehending intricate financial and physical systems, but it also opens up fresh possibilities for solving real-world issues that involve intrinsic uncertainty and randomness. The spectral method is a flexible and reliable method because it can effectively manage the fractional-order derivatives in FSDDEs and capture the underlying spectrum features of the processes involved. Additionally, it is anticipated that ongoing development of computational tools and software packages designed for spectral-based FSDDEs solutions would improve this approach’s usability and accessibility, democratizing its applicability across various scientific and industrial disciplines.

## Data Availability

The datasets used and/or analysed during the current study available from the corresponding author on reasonable request.
